# Salicylate-induced changes in immediate-early genes in the hippocampal CA1 area

**DOI:** 10.3892/mmr.2015.3608

**Published:** 2015-04-14

**Authors:** HAO WU, FENG-LEI XU, YONG YIN, PENG DA, XIAO-DONG YOU, HUI-MIN XU, YAN TANG

**Affiliations:** 1 Department of Otolaryngology-Head and Neck Surgery, Affiliated Hospital of Nantong University, Nantong, Jiangsu 226001, P.R. China; 2 Department of Otolaryngology-Head and Neck Surgery, Nanjing General Hospital of Nanjing Military Area, Nanjing, Jiangsu 210002, P.R. China; 3 Department of Otolaryngology, Changshu First Hospital, Suzhou, Jiangsu 215500, P.R. China

**Keywords:** salicylate, hippocampus, activity-regulated cytoskeleton-association protein 3.1, early growth response gene 1, *N*-methyl D-aspartate receptor subunit 2B

## Abstract

Studies have suggested that salicylate affects neuronal function via interactions with specific membrane channels/receptors. However, the effect of salicylate on activity and synaptic morphology of the hippocampal *Cornu Ammonis* (CA) 1 area remains to be elucidated. The activation of immediate-early genes (IEGs) was reported to correlate with neuronal activity, in particular activity-regulated cytoskeleton-associated protein and early growth response gene 1. The aim of the present study was to evaluate the expression of these IEGs, as well that of *N*-methyl D-aspartate (NMDA) receptor subunit 2B in rats following acute and chronic salicylate treatment. Protein and messenger RNA levels of all three genes were increased in rats following chronic administration of salicylate (300 mg/kg for 10 days), returning to baseline levels 14 days post-cessation of treatment. The transient upregulation of gene expression following treatment was accompanied by ultrastructural alterations in hippocampal CA1 area synapses. An increase in synaptic interface curvature was observed as well as an increased number of presynaptic vesicles; in addition, postsynaptic densities thickened and lengthened. In conclusion, the results of the present study indicated that chronic exposure to salicylate may lead to structural alteration of hippocampal CA1 neurons, and it was suggested that this process occurs through induced expression of IEGs via NMDA receptor activation.

## Introduction

Salicylate is a widely prescribed compound known for its anti-pyretic, anti-inflammatory and analgesic properties (1). However, salicylate has been reported to have numerous undesirable side effects, including gastrointestinal irritation and bleeding, tinnitus, hypersensitivity as well as central nervous system (CNS) symptoms (2). It has been suggested that salicylate affects neuronal function via interactions with specific membrane channels/receptors (3); however, the majority of studies to date have focused on its effects on the auditory system (4–6). Salicylate treatment was reported to increase spontaneous neuronal activity and decrease the efficacy of inhibitory neurotransmission in the inferior colliculus and auditory cortex (7,8). However, the effect of salicylate on neural plasticity in the hippocampus, a key structure for numerous complex brain functions, including exploration, cognition, and memory, remains to be elucidated (9).

Tinnitus is the perception of sound in the absence of an external acoustic stimulus. It is not clear whether chronic tinnitus can be solely attributed to damage to the auditory system; of note, an association between tinnitus and emotional state has been reported (10–13), indicating that tinnitus may also result from the dysfunction of non-auditory brain regions such as the hippocampus (14). The hippocampus receives either direct or indirect input from the central auditory system (15), which in turn receives projections from limbic regions that modulate neuronal activity and plasticity (16).

Long-term plasticity is dependent on rapid, *de novo* protein synthesis. Immediate-early genes (IEGs), including activity-regulated cytoskeleton-associated protein (Arc/Arg) 3.1 and early growth response gene 1 (Egr-1), were reported to have major roles in transcription-dependent plasticity (17). The aim of the present study was to evaluate the expression of these genes, as well as that of *N*-methyl D-aspartate (NMDA) receptor subunit 2B (NR2B), in order to measure the neuronal activity and morphology of neurons in the hippocampal CA1 area of rats following chronic exposure to salicylate.

## Materials and methods

### Animals

Experimental procedures were approved by the Animal Care and Use Committee of the Nantong University School of Medicine (Jiangsu, China). A total of 54 male Sprague-Dawley rats (Shanghai Super-B&K Laboratory Animal Corp. Ltd., Shanghai, China), aged 2–3 months (weight, 250–350 g) were divided into five groups: (1) Control group (n=12); (2) acute treatment group administered a single salicylate injection (n=6); (3) chronic treatment group (S10) administered daily injections of salicylate for 10 days (n=12); (4) recovery group (S10+R14) with a 14-day recovery period following chronic salicylate treatment (n=6); and (5) recovery group (S10+R28) with a 28-day recovery period following chronic salicylate treatment (n=9).

### Experimental design and salicylate administration

Sodium salicylate (Sigma-Aldrich, Shanghai, China) was dissolved in normal saline [9% (w/v) NaCl] for a final concentration of 100 mg/ml. Rats in the acute treatment group received a single intraperitoneal (i.p.) injection of salicylate (300 mg/kg). Rats were then anesthetized using sodium pentobarbital (40 mg/kg, i.p.; P3761, Sigma-Aldrich, St. Louis, MO, USA) and sacrificed 2 h post-treatment. Animals in the chronic treatment groups were administered i.p. injections of salicylate daily at 08:00 h for 10 consecutive days. Rats in the S10 group were sacrificed at 08:00 h on day 11, while those in S10+R14 and S10+R28 groups were sacrificed at 08:00 h on days 25 and 39, respectively. Animals in the control group were administered an i.p. injection of saline at 08:00 h for 10 consecutive days.

### Reverse transcription quantitative PCR (RT-qPCR)

Rats were sacrificed by decapitation following an i.p. injection of sodium pentobarbital (40 mg/kg body weight). The CA1 region of the hippocampus was immediately dissected and total RNA was extracted using TRIzol reagent (Invitrogen Life Technologies, Carlsbad, CA, USA) according to the manufacturer’s instructions. RNA was quantified by measuring absorbance at 260/280 nm (TENOVO International Co., Ltd., Beijing, China) and complementary DNA (cDNA) was obtained using a Reverse Transcription kit (DRR036A; Takara Bio Inc., Otsu, Japan). Primers for Arg3.1, Egr-1, NR2B and GAPDH were obtained from Shanghai Sangon Biological Engineering Technology and Services Co., Ltd. (Shanghai, China). Primer sequences were as follows: Arc forward (F), 5′-CTGCCACAGAAGCAGGGTGA-3′ and Arc reverse (R), 5′-AGGGTGCCCACCACATACTGA-3′; Egr-1-F, 5′-GAACAACCCTACGAGCACCTG-3′ and Egr-1-R, 5′-GCCACAAAGTGTTGCCACTG-3′; NR2B-F, 5′-TGGCTATCCTGCAGCTGTTTG-3′ and NR2B-R, 5′-TGGCTGCTCATCACCTCATTC-3′; and GAPDH-F, 5′-GGCACAGTCAAGGCTGAGAATG-3′ and GAPDH-R, 5′-ATGGTGGTGAAGACGCCAGTA-3′. SYBR Premix Ex Taq (DRR420A; Takara Bio, Inc., Otsu, Japan) was used as the reaction mixture.

The cycling program was as follows: 95°C for 30 sec; 40 cycles of 95°C for 5 sec, 60°C for 34 sec; and a final dissociation stage using the ABI 7500 real-time PCR system (Applied Biosystems, Foster City, CA, USA). The amplification efficiency of the target and reference (GAPDH) were assumed to be equal. Relative quantification and calculations were performed using the comparative threshold (Ct) cycle method (2^−ΔΔCt^) (18).

### Western blot analysis

Total protein was extracted from tissue samples and the concentration was determined using an ultraviolet spectrophotometer (DR/4000UV-VIS; Hach, Loveland, CO, USA). SDS-PAGE was performed using a 12% polyacrylamide gel to resolve Arg3.1 and Egr-1, while 8% polyacrylamide was used for NR2B. Proteins were transferred to polyvinylidene difluoride membranes, which were incubated in blocking buffer (Tris-buffered saline, containing 0.1% Tween-20 and 5% skimmed milk powder), and then incubated with primary antibodies diluted in the same buffer overnight at 4°C. Following washes in Tris-buffered saline with 0.1% Tween-20, membranes were incubated with secondary antibodies in blocking buffer for 2 h at room temperature. The primary antibodies used were rabbit polyclonal anti-Arc (1:1,000, ab23382; Abcam, Cambridge, MA, USA), rabbit anti-Egr-1 (1:1000, 4153S; Cell Signaling Technology, Danvers, MA, USA) and rabbit anti-NR2B (1:1,000, 4212S; Cell Signaling Technology); followed by the secondary antibody goat anti-rabbit immunoglobulin G horseradish peroxidase (1:5,000; Jackson ImmunoResearch, Inc., West Grove, PA, USA). Immunoreactivity was visualized using the SuperSignal West Pico Chemiluminescent Substrate system (Pierce Biotechnology, Inc., Rockford, IL, USA). Band intensity was quantified using Image Lab software version 3.0 (Bio-Rad, Hercules, CA, USA), and values for Arc, Egr-1 and NR2B are expressed relative to GAPDH.

### Immunohistochemistry

Rats were anesthetized using 2% sodium pentobarbital and perfused through the ascending aorta with normal saline followed by 4% (v/v) paraformaldehyde. The brain was removed and the hippocampal CA1 region was dissected, then embedded in paraffin. Paraffin blocks were sectioned at 10 *µ*m, sections were then collected on slides and deparaffinized using xylene prior to rehydration through a graded alcohol series. Sections were washed in phosphate-buffered saline (PBS; pH 7.4) with 0.05% Tween-20 (PBST) and then blocked with 1% normal goat serum in PBS for 30 min at 37°C. Consecutive sections were incubated with one of the following primary antibodies: rabbit anti-Arc (1:50, ab23382; Abcam), rabbit anti-Egr-1 (1:50, 4153S; Cell Signaling Technology) or rabbit anti-NR2B (1:50, 4212S; Cell Signaling Technology). Sections were rinsed with PBST and incubated with horseradish peroxidise-conjugated secondary antibody (goat anti-rabbit IgG, 1:2,000; Sigma-Aldrich, Gillingham, UK) for 1 h at room temperature. Immunoreactivity was visualized by treating the sections with 0.015% (v/v) H_2_O_2_ in 3,3′-diaminobenzidinetetrahydrochloride/Tris-buffered saline for 10 min at room temperature.

Coronal sections of the hippocampal CA1 region were visualized using an Axioplan 2 imaging microscope (Carl Zeiss Microimaging Inc., Thornwood, NY, USA) and analyzed using ImageJ software version 1.48 (National Institutes of Health, Bethesda, MD, USA) to quantify the density of immunoreactive neurons as the number of positive neurons/section. Hippocampal neurons from the same side of the brain were counted for each sample.

### Transmission electron microscopy (TEM)

In order to determine whether changes in IEG expression were due to, or produced by, alterations in synaptic properties, hippocampal CA1 neurons of nine animals (three each from the control, S10 and S10+R28 groups) were examined using TEM. Anesthetized rats were perfused via the ascending aorta with 2% (v/v) glutaraldehyde (Sigma-Aldrich) in saline. Following perfusion, brains were removed and the hippocampus was dissected, then washed in 0.1 M phosphate buffer. Tissue samples were immersed in 2% glutaraldehyde and 1% osmium tetroxide (Sigma-Aldrich) for 2 h at 4°C, then dehydrated in a graded ethanol series. Following displacement of ethanol with propylene oxide (Sigma-Aldrich), the tissue was embedded in Epon (Sigma-Aldrich) and sectioned along the coronal plane with a diamond knife (FernAnclez-hIorln 1953; Ivan Sorvall, Inc., New York, NY, USA) at a thickness of 70 *µ*m. The sections were stained with lead citrate and observed using a CM-120 electron microscope (Philips, Eindhoven, Netherlands). ImageJ software was used for quantitative analysis of three sections in the superficial layers of the hippocampal CA1 region, performed separately for each hemisphere (19). The number of synaptic vesicles, thickness of the postsynaptic density (PSD), width of the synaptic cleft and curvature of the synaptic interface were measured (20).

### Statistical analysis

Statistical analyses were performed using SPSS version 19 (SPSS, Inc., Chicago, IL, USA) and all data are presented as the mean ± standard deviation. Based on the distribution of data and homogeneity of variance, the unpaired, two-tailed Student’s t-test and one-way analysis of variance followed by Dunnett’s post-hoc tests were used to compare the results from each group. P<0.05 was considered to indicate a statistically significant difference between values.

## Results

### Expression of IEGs in the hippocampal CA1 region

**Figure 1:**
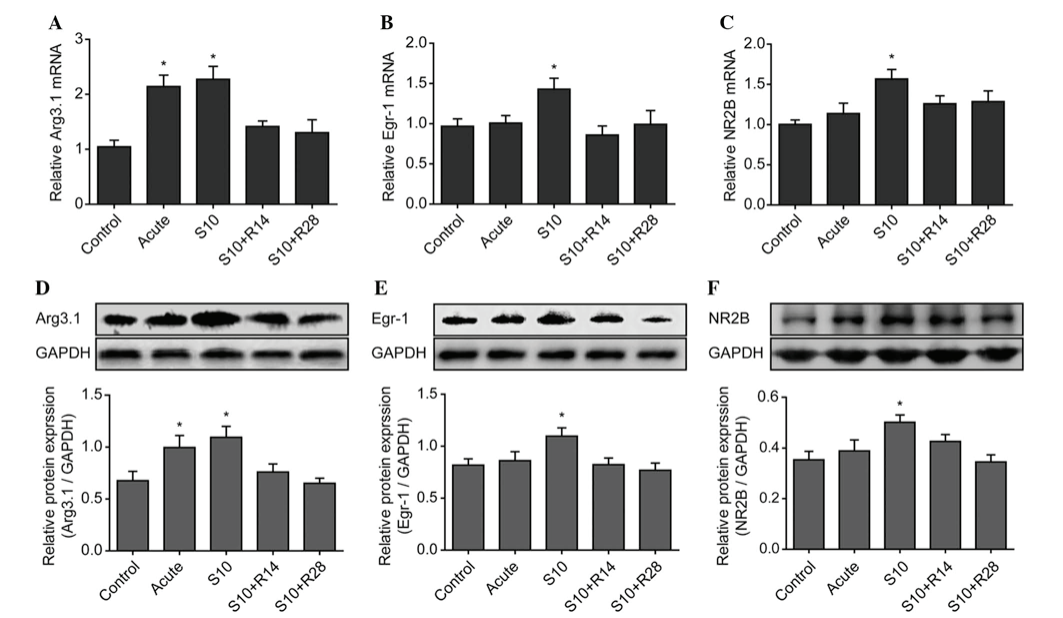
Effect of salicylate administration on the expression of Arg3.1, Egr-1, and NR2B mRNA and protein in the hippocampal CA1 area. mRNA expression levels for (A) Arg3.1, (B) Erg-1 and (C) NR2B were quantified using reverse transcription quantitative polymerase chain reaction. Transcript levels were normalized to GAPDH protein expression levels of (D) Arg3.1, (E) Erg-1 and (F) NR2B were quantified using western blot analysis. GAPDH was used as a loading control. Arg3.1 expression was upregulated in acute and S10 groups compared to that of the control groups. Similarly, increased expression of Egr-1 and NR2B was observed in animals in the S10 group. ^*^P<0.05 compared to controls. Arg3.1, activity-regulated cytoskeleton-associated protein; Egr-1, early growth response gene 1; NR2B, *N*-methyl D-aspartate (NMDA) receptor subunit 2B; CA1, *Cornu Ammonis* 1; mRNA, messenger RNA; IEG, immediate-early genes; S10, chronic salicylate treatment group; R#, number of recovery days.

RT-qPCR and western blot analysis were used to evaluate the expression of IEGs in the hippocampal CA1 region following treatment with salicylate. mRNA and protein expression of Arg 3.1 were upregulated in rats subjected to acute and chronic (S10) salicylate exposure (P<0.05) (Fig. 1). By contrast, levels in the S10+R14 and S10+R28 groups demonstrated no significant difference from those of controls. For Egr-1 and NR2B, mRNA and protein levels were upregulated in the S10 group; however, the two recovery groups showed no significant difference compared to those of the control animals (P<0.05). These results therefore indicated that gene expression returned to baseline levels following cessation of salicylate treatment.

**Figure 2:**
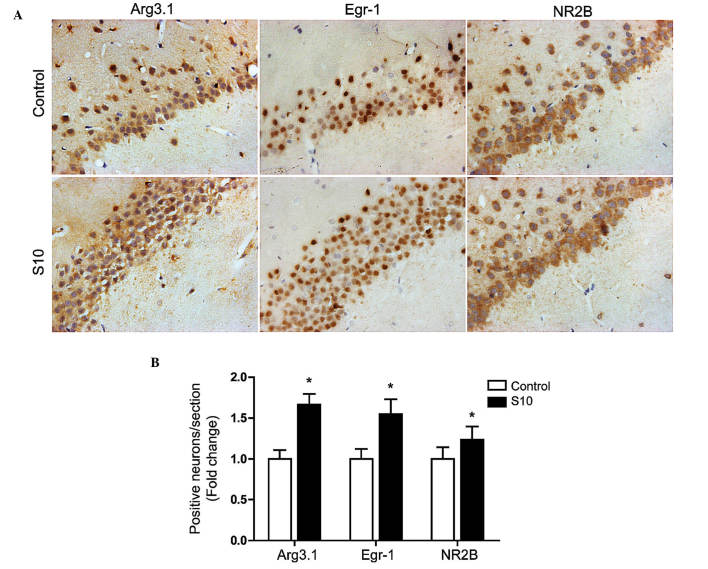
Immunohistochemical analysis of expression of Arg3.1, Egr-1 and NR2B in hippocampal *Cornu Ammonis* 1 neurons following salicylate administration. (A) Coronal sections of the hippocampi of rats receiving S10 and saline-injected controls were labelled with antibodies against Arg3.1, Egr-1, and NR2B (magnification, x400). (B) Number of cells expressing each of the three markers in S10 group, represented as a fold change of the control group. ^*^P<0.05 compared to controls. Arg3.1, activity-regulated cytoskeleton-associated protein; Egr-1, early growth response gene 1; NR2B, *N*-methyl D-aspartate receptor subunit 2B; S10, chronic salicylate treatment group.

In addition, immunohistochemical examination of IEG expression in the hippocampal CA1 region revealed an increased number of Arg3.1-, Egr-1- and NR2B-positive neurons in the S10 group compared with that of the control group (Fig. 2).

### Ultrastructural alterations of synapses

**Figure 3:**
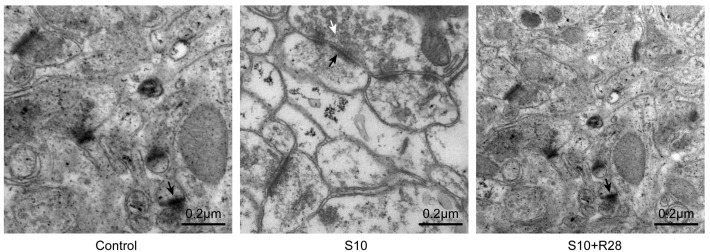
Ultrastructural alterations in synapses of hippocampal *Cornu Ammonis* 1 neurons in salicylate-treated animals. Representative images are shown at magnification, ×30,000. Transmission electron micrographs show that animals in the S10 group had a greater number of presynaptic vesicles (white arrows), thicker postsynaptic densities (black arrows), and greater synaptic interface curvature, as well as fewer microtubules and neurofilaments (arrowheads), than saline-injected control animals. S10, chronic salicylate treatment group; R#, number of recovery days.

Examination of synaptic ultrastructure of hippocampal CA1 neurons revealed an increased number of synaptic vesicles (P<0.05), thicker PSD (P<0.05) and increased synaptic interface curvature (P<0.05) in the S10 group compared to those of the control animals (Fig. 3 and Table I).

In conclusion, these results demonstrated that salicylate induced the upregulation of IEG expression and resulted in physical alterations to the synaptic structure of hippocampal CA1 neurons that persisted for the duration of salicylate exposure.

## Discussion

As one of the most commonly used non-prescription drugs on the market, salicylate and its derivative aspirin, are able to penetrate the blood-brain barrier; with cerebrospinal concentration of salicylate reaching several millimolars in an animal model (21). The results of the present study demonstrated that long-term administration of salicylate within this concentration range induces the expression of Arg3.1, Egr-1, and NR2B. Given the roles of the NMDA receptor in synaptic plasticity and the role of Arg3.1 in cytoskeletal remodeling (22), the observed ultrastructural changes at hippocampal CA1 neuronal synapses in the salicylate treatment group indicated that these morphological changes may be a direct consequence of IEG upregulation.

High-intensity noise was reported to induce hippocampal plasticity and the symptoms of tinnitus by altering the response of place cells (23). However, whether salicylate induces plasticity in the hippocampus remains to be elucidated, although this effect has been observed in the peripheral and central auditory systems (24). Upregulation of NR2B in the forebrain of transgenic mice was reported to be associated with the activation of NMDA receptors, which are critical for regulating the age-dependent thresholds of plasticity and memory formation (25). Egr-1 was found to be essential for the persistence of late-phase long-term potentiation in the hippo-campus and the consolidation of several forms of long-term memory (26); Arg3.1 was reported to have comparable roles in long-term memory consolidation and synaptic plasticity (27). Arg3.1 acts as an effector protein at synapses; Arg3.1 mRNA is trafficked to dendrites and accumulates at sites of synaptic activity, where it is locally translated and induces homeostatic scaling of α-amino-3-hydroxy-5-methyl-4-isoxazolepropionic acid receptors and structural modifications (28). Therefore, the results of the present study, which demonstrated the upregulation of Arg3.1, Egr-1 and NR2B in the hippocampus following chronic salicylate administration, reflect, in part, the establishment of long-term plastic changes.

Salicylate was reported to inhibit cochlear cyclooxygenase and stimulate arachidonic acid production, which facilitated the NMDA receptor response to glutamate released spontaneously by inner hair cells (29). In a fear memory consolidation paradigm, NMDA receptor-mediated alterations in extracellular signal-regulated kinase/mitogen-activated protein kinase signaling promoted the transcription and translation of the IEG Egr-1 in neurons of the lateral nucleus of the amygdale (30). Egr family members regulate Arg3.1 transcription, and Egr-1 activation modulate later phases of activity-dependent Arg3.1 transcription in the dentate gyrus and CA1 area of the hippocampus (31). This therefore indicated that the observed ultrastructural changes resulting from salicylate treatment were due to NMDA receptor-mediated activation of Egr-1, followed by upregulation of Arg3.1 expression and remodeling of cytoskeletal components.

To date, few studies have investigated the time course of changes occurring in the hippocampus following salicylate exposure. The present observation that Arg3.1 levels significantly increased in the acute as well as the chronic treatment groups suggested that Arg3.1 acts immediately to fine-tune the neuronal response to activity. However, the transcript and protein levels of NR2B, Egr-1 and Arg3.1 returned to normal 14 days post-cessation of salicylate treatment, consistent with the reversible increases in cochleoneural activity (32), distortion product otoacoustic emissions (33), and cochlear prestin expression (34) in the auditory system reported by previous studies. These fluctuations indicated a homeostatic mechanism through which the nervous system adapts to novel stimulation, providing a basis for long-term plasticity.

Salicylate-treated rats had a greater number of presynaptic vesicles, thicker PSDs and increased synaptic interface curvature. This suggested that synapses are primed for increased neurotransmitter release and synaptic transmission, which may results in greater synaptic efficacy. The observations were comparable with ultrastructural changes reported by a previous study, which found large, lucent pleomorphic vesicles in the synapses of the dorsal cochlear nucleus of the chinchilla following acoustic trauma (35). Of note, images from the TEM analysis showed that there were fewer microtubules and neurofilaments in the synapses of salicylate-treated animals, which may possibly lead to reduced vesicular and protein transport to the synapse. Further studies are required to determine whether this was a compensatory reduction in response to salicylate-induced hyperactivity.

In conclusion, the results of the present study demonstrated that chronically administered salicylate produced transient changes in the expression of IEGs as well as the synaptic ultrastructure in hippocampal CA1 neurons. Future studies are required in order to investigate whether these changes are associated with tinnitus in these animals by employing the gap-startle paradigm, which could ultimately provide insight into the role of the hippocampus in the development of tinnitus in humans.

## Figures and Tables

**Table I:** Synaptic parameters for the different experimental groups.

Synaptic parameters	Controls	S10	S10+R28
Vesicles (number/*µ*m^2^)	5±3	73±24^b^	6±3
Cleft width (*µ*m)	0.0083±0.0023	0.0088±0.0023	0.0082±0.0016
Postsynaptic density thickness (*µ*m)	0.035±0.008	0.048±0.009^a^	0.038±0.011
Curvature	0.63±0.22	0.95±0.21^a^	0.68±0.26

Mean ± standard deviation for each parameter (n=18). The control group received daily intraperitoneal injections of saline for 10 consecutive days; the S10 group received daily intraperitoneal injections of salicylate (300 mg/kg) for 10 days; the S10+R28 group underwent identical treatment to that of the S10 animals, but were allowed to recover for 28 days before they were sacrificed for analysis.

a P<0.05;

b P<0.001 compared to control. S10, chronic salicylate treatment group; R#, number of recovery days.
